# Microbial agents in macroscopically healthy mammary gland tissues of small ruminants

**DOI:** 10.7717/peerj.3994

**Published:** 2017-11-13

**Authors:** Liliana Spuria, Elena Biasibetti, Donal Bisanzio, Ilaria Biasato, Daniele De Meneghi, Patrizia Nebbia, Patrizia Robino, Paolo Bianco, Michele Lamberti, Claudio Caruso, Alessia Di Blasio, Simone Peletto, Loretta Masoero, Alessandro Dondo, Maria Teresa Capucchio

**Affiliations:** 1 Department of Veterinary Sciences, University of Turin, Grugliasco, Italy; 2 Big Data Institute, Nuffield Department of Medicine, University of Oxford, c/o Wellcome Trust Centre for Human Genetics, Oxford, United Kingdom; 3 Sacro Cuore Don Calabria Hospital, Negrar, Verona, Italy; 4 ASLTo4, ASL, Torino, Italy; 5 ASLCn1, ASL, Barge, Italy; 6 Istituto Zooprofilattico Sperimentale di Piemonte, Liguria e Valle d’Aosta, Torino, Italy

**Keywords:** Small ruminants, Microbiology, Histology, Healthy mammary gland

## Abstract

**Background:**

Health of mammary glands is fundamental for milk and dairy products hygiene and quality, with huge impacts on consumers welfare.

**Methods:**

This study aims to investigate the microbial agents (bacteria, fungi and lentiviruses) isolated from 89 macroscopically healthy udders of regularly slaughtered small ruminants (41 sheep, 48 goats), also correlating their presence with the histological findings. Multinomial logistic regression was applied to evaluate the association between lesions and positivity for different microbial isolates, animal age and bacteria.

**Results:**

Twenty-five samples were microbiologically negative; 138 different bacteria were isolated in 64 positive udders. Coagulase-negative staphylococci were the most prevalent bacteria isolated (46.42%), followed by environmental opportunists (34.76%), others (10.14%) and pathogens (8.68%). Most mammary glands showed coinfections (75%). Lentiviruses were detected in 39.3% of samples. Histologically, chronic non-suppurative mastitis was observed in 45/89 glands, followed by chronic mixed mastitis (12/89) and acute suppurative mastitis (4/89). Only 28 udders were normal. Histological lesions were significantly associated with the animal species and lentiviruses and coagulase-negative staphylococci infections. Goats had significantly higher risk to show chronic mixed mastitis compared to sheep. Goats showed a significantly lower risk (OR = 0.26; 95% CI [0.06–0.71]) of being infected by environmental opportunists compared to sheep, but higher risk (OR = 10.87; 95% CI [3.69–37.77]) of being infected with lentiviruses.

**Discussion:**

The results of the present study suggest that macroscopically healthy glands of small ruminants could act as a reservoir of microbial agents for susceptible animals, representing a potential risk factor for the widespread of acute or chronic infection in the flock.

## Introduction

The importance of small dairy ruminants has significantly increased during last decades, especially in developing countries where they represent fundamental economic and social livelihoods of a large human population (Oluwatayo & Oluwatayo, 2012). In high income economies, they are also becoming an important alternative to supply dairy products for human consumption (Lérias et al., 2014).

Functionality and health of the mammary gland are fundamental for dairy farms and directly correlated with milk production, milk and dairy products hygiene and quality, and consequently farmers profits. Mastitis is the inflammation of the mammary gland characterized by an increase in the number of somatic cells in the milk and/or by pathological changes in the mammary tissue (Schlafer & Foster, 2016). It is generally caused by microbial agents invasion, mainly represented by bacteria, and in a small extent by mycoplasmas, viruses (Small Ruminant Lentivirus—SRLV), fungi and algae.

According to Bergonier & Berthelot (2003a), mastitis is important from three perspectives. Firstly, there are economic implications, because of reduced growth of lambs and kids and their mortality, treatment costs, reduced milk production and milk prices dependent on cellular quality in particular areas. Furthermore, hygienic issues related to the risk of infection or intoxication of consumers by milk bacteria such as *Escherichia coli*, *Staphylococcus aureus*, *Listeria monocytogenes*, *Salmonella* spp., etc can occur. Finally, the E.U. Directive 46/92 (modified by Directive 71/94), which defines the milk bacteriological quality, must be considered (Bergonier et al., 2003b; Olechnowicz & Jaśkowski, 2014). In sheep, mammary infections are also of great welfare concern (European Food Safety Authority, 2009), since clinical mastitis is a disease that leads to anxiety, restlessness, changes in feeding behavior and pain in affected ewes (Fthenakis & Jones, 1990). Even in subclinical mastitis, normal behavioral patterns of sheep are modified (Gougoulis et al., 2008; Gougoulis, Kyriazakis & Fthenakis, 2010), hence raising potential welfare concerns (Gelasakis et al., 2015).

Mastitis of small ruminants are commonly divided into clinical and subclinical forms. Clinical mastitis is the term used for inflammatory process of the mammary gland, mostly from bacterial infection, which is accompanied by overt clinical symptoms. These visible signs of clinical mastitis include swelling, redness or necrosis of one or more half udders and/or abnormal discharge of milk. Affected animals can also present with anorexia, fever or agalactia. Progression of clinical mastitis might be toxaemia and gangrenous necrosis of the udder (Omaleki et al., 2011), which could result in a fatal event. Conversely, subclinical mastitis is characterized by inflammation of the udder that can be diagnosed only by enumeration of inflammatory cells in milk or inflammatory markers evaluation (White & Hinckley, 1999; Olechnowicz & Jaśkowski, 2014; Addis et al., 2016; Bramis et al., 2016). Among the organisms associated with clinical mastitis, *Staphylococcus aureus* has been reported to be the most common in both meat and dairy ewes milk samples. *Mannheimia haemolytica*, *Escherichia coli* and various streptococci are other important causative organisms isolated from udder secretions (Mørk et al., 2007; Smith et al., 2015; Arsenault et al., 2008; Dore et al., 2016). Coagulase-negative staphylococci (CNS) are the most prevalent bacteria isolated in subclinical mastitis of sheep and goats (Contreras et al., 2007; Ergün et al., 2009; Onni et al., 2010; Addis et al., 2016; Bramis et al., 2016; Dore et al., 2016), with a prevalence in dairy ewes ranging from 25 to 93%. On the contrary, they are less frequently observed in meat ewes (12–34%) (Olechnowicz & Jaśkowski, 2014). Small Ruminant Lentivirus infection has also been reported to be an important cause of chronic subclinical mastitis predisposing the gland to secondary infections (Cutlip et al., 1985; Brodie et al., 1995; Arsenault et al., 2003; Arsenault et al., 2008).

While the mammary gland is the specialized milk production organ, no studies about the microbiological flora isolated from healthy mammary gland tissue of small ruminants and, particularly, the correlation between microbiological data and histological findings are currently available.

For this reason the aim of the present study was to investigate the microbial agents (bacteria, fungi and SRLV) isolated from macroscopically healthy mammary parenchyma of regularly slaughtered small ruminants and to correlate their presence with the histological findings.

## Materials & Methods

### Sample collection

A total of 89 macroscopically healthy small ruminants udders (*n* = 41 sheep—23 lactating and 18 non-lactating—and *n* = 48 goats—36 lactating and 12 non-lactating) belonging to a total of 25 ovine and 27 caprine flocks, respectively, were collected between October 2013 and February 2016 in two slaughterhouses of northern Italy, Piedmont region. Animals enrolled in the study were slaughtered for different causes unrelated to the mammary glands involvement.

The sheep ranged in age from one to 15 years (median five) and were mainly Biellese (*n* = 20; 48.8%) and cross-breed (*n* = 17; 41.5%), with Frabosana (*n* = 2; 4.9%), Lacaune (*n* = 1; 2.4%) and Sambucana breed (*n* = 1; 2.4%) being less represented. The goats ranged in age from 2 to 13 years (median 5.5) and were mainly Saanen (*n* = 13; 27.1%) and cross-breed (*n* = 25; 52.1%), with a lower prevalence of Camosciata delle Alpi breed (*n* = 10; 20.8%). Animals were classified in three groups according to their age: the first group included 27 animals which were up to three years old (*n* = 14 sheep and *n* = 13 goats), the second group was composed of 44 animals aged from four to eight years (*n* = 20 sheep and *n* = 24 goats) and the third group was composed of 18 animals over eight years (*n* = 7 sheep and *n* = 11 goats).

Only udders considered free of clinical mastitis and any other alterations at gross examination (inspection and palpation for macroscopic signs of abnormalities) were included in this study.

After gross examination, all the udders were stored at 4 °C and transported to the laboratory of the Department of Veterinary Sciences of University of Turin. The skin of the udder was washed, dried and disinfected with cotton soaked with 70% alcohol. Two fragments of parenchyma of approximately 2.5 cm^3^, were collected in two different points and fixed in vials containing 10% neutral buffered formalin for histopathological examination, one sample was frozen at −20 °C as tissue bank, and the remaining tissue was sent to the Istituto Zooprofilattico Sperimentale of Piemonte, Liguria and Valle d’Aosta (Turin) for bacteriological, mycological and virological investigations.

### Bacteriology

The udder surface was sterilized through cauterization and then cut using scalpel until the glandular tissue was exposed. A swab was inserted into the incision and then it was streaked on several agar plates. Columbia agar containing 5% sheep blood (Liofilchem, Roseto degli Abruzzi, Italy) and Chocolate agar (Liofilchem, Roseto degli Abruzzi, Italy) plates were used and incubated at 37 °C aerobically and 5% CO^2^-enriched atmosphere, respectively, for 24–48 h. Bacteria identification was carried out by phenotypic identification methods; for this purpose a growth-based automatic methods (Vitek 2 System; bioMérieux, Marcy-l’Étoile, France) that measures either turbidity or colored products by various metabolic activities such as acidification, alkalinization, enzyme hydrolysis, and growth in the presence of inhibitory substances was used. According to Carter & Cole (1990) it has been selected only the colonies numerically representative, in pure culture or showing phenotypically features as haemolysis. In order to obtain a clear interpretation of the results, a parallel control for the identification of inhibiting substances in the udders was performed. Therefore, two fragments of parenchyma were placed on an agar plate containing an inoculum of *Bacillus subtilis* and incubated aerobically for 24 h at 37 °C. The test was considered positive if a ring around the fragment, which indicates the inhibition of bacterial growth, was observed.

For the detection of *Mycoplasma* spp., a PPLO Selective Agar (Microbiol, Sardinia, Italy) was incubated at 37 °C in 5% CO^2^-enriched atmosphere. A pre-enrichment was performed in order to promote the isolation of *Mycoplasma* spp. In particular, a fragment of parenchyma was inoculated in PPLO Broth (Microbiol, Sardinia, Italy) and incubated at 37 °C in 5% CO^2^-enriched atmosphere. After 72 h, 0.1 ml of broth was inoculated on PPLO agar. Mycoplasma’s growth was monitored for 10 days by microscopic examination with the aim to identify any typical tiny “fried egg” or finely granular (“ground glass”) colonies that penetrate the agar surface.

Bacteria were classified in four groups:

• pathogens (PATO): according to the literature, pathogenic bacteria reported in clinical mastitis;
• coagulase-negative staphylococci (CNS): opportunistic bacteria reported in the normal skin microbial flora usually responsible for subclinical mastitis. Their pathogenicity is highly variable from asymptomatic to mild forms. Sporadic cases of clinical mastitis, especially in immunocompromised animals, are also described;
• environmental opportunists (EO): ubiquitous microorganisms detected in the gastro-enteric tract, soil, water and bedding. Once penetrated within the udder, they are able, under particular conditions, to become pathogenic and cause mastitis of different severity;
• other microorganisms (OM): bacteria not isolated in clinical or subclinical mastitis.

### Mycology

Sabouraud agar plate (Biolife, Bolzano, Italy) was used for the isolation of several types of fungi and filamentous bacteria, directly streaking the swab, as previously described, on the surface on the plate. The plate was incubated for 10 days at room temperature. In case of growth of suspect colonies, a microscopic examination was carried on in order to obtain an identification at genus-level.

### Virology

For SRLV detection, DNA was extracted from 50–100 mg of udder tissue by using ReliaPrep™ gDNA Tissue Miniprep System (Promega, Madison, WI, USA), following manufacturer’s instructions. DNA was amplified following a slightly modified version of the protocol described in Grego et al. (2007). Briefly, two forward degenerate primers, GAG F1 (5′-TGGTGARKCTAGMTAGAGACATGG-39) and GAG F2 (5′-CAAACWGTRGCAATG CAGCATGG-3′), and two reverse degenerate primers POL R1 (5′-CATAGGRGGHGCGGA CGGCASCA-3′) and POL R2 (5′-GCGGACGGCASCACACG-3′) were selected, based on conserved regions (Grego et al., 2007). This primer set allows to detect all the different SRLV genogroups: SRLV group A (MVV—prototype-like strains), SRLV group B (CAEV—prototype-like strains) and also SRLV group E (Roccaverano strains), a specific genogroup circulating in the studied area.

The first PCR reaction was carried out in a total volume of 50 µl containing 1X PCR Buffer (Platinum Taq DNA Polymerase; Invitrogen, Carlsbad, CA, USA), 0.2 mM of dNTPs, 3 mM of MgCl_2_, 0.3 µM of each primers, 1U Taq Polymerase (Invitrogen, Carlsbad, CA, USA). The PCR was performed under the following conditions: initial template denaturation step at 95 °C for 15 min; 35 cycles of denaturation at 94 °C for 30 s, annealing at 55 °C for 1 min, and elongation at 72 °C for 2 min, followed by a final elongation step at 72 °C for 10 min. Second PCR was carried out in the same conditions (using primers GAG F2 and POL R2), with a lower annealing temperature (55 °C) for a total of 45 cycles. Amplification products were loaded and checked on a 2% agarose gel. Moreover, to reach 100% specificity, all nested PCR amplicons of expected sizes were directly sequenced on an ABI 3130 genetic analyzer (Life Technologies, Carlsbad, CA, USA) and generated sequences were submitted to a Blast search for confirmation.

### Histology

**Figure 1:**
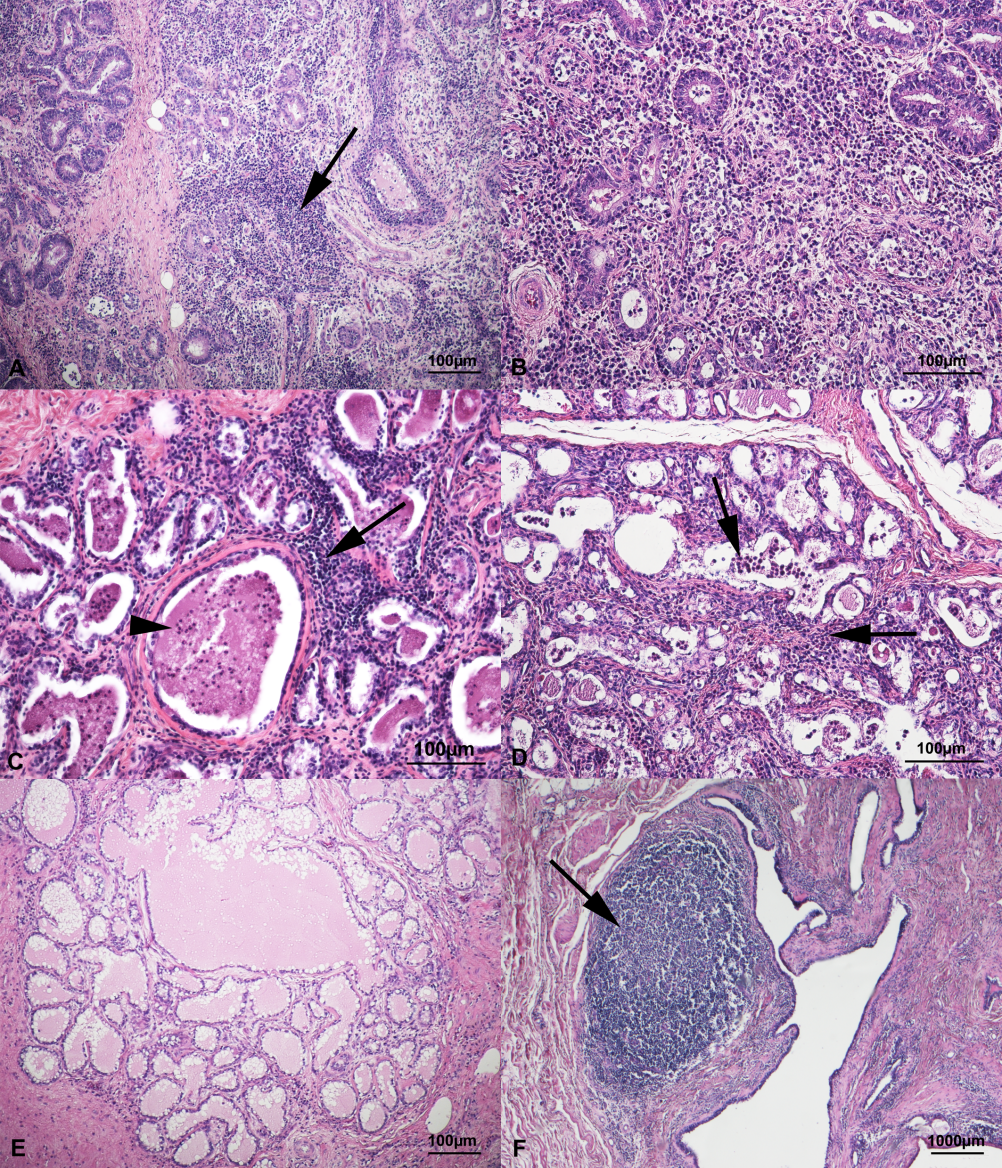
Histological findings in healthy mammary glands of small ruminants. (A) Chronic non suppurative mastitis characterized by interstitial (arrow) and alveolar mononuclear inflammatory cells (lymphocytes, plasmacells and macrophages). Haematoxylin & Eosin (H&E) stain. (B) Particular of the mononuclear inflammation in chronic non-suppurative mastitis. H&E stain. (C) Chronic mixed mastitis with interstitial mononuclear inflammatory cells (arrow) and alveolar neutrophils (arrowhead). H&E stain. (D) Acute suppurative mastitis characterized by interstitial and alveolar neutrophils (arrows). H&E stain. (E) A normal mammary tissue. H&E stain. (F) Small Ruminant Lentiviruses positive mammary gland with wide and focal hyperplastic lymphoid follicle (arrow) around a lactiferous duct. H&E stain.

Formalin fixed fragments of parenchyma were paraffin embedded and cut at 5 µm thickness sections. The sections were stained with haematoxylin and eosin (H&E) and examined by light microscope at different magnifications. Microscopically, lesions were classified as negative when they showed a normal pattern; affected by chronic non-suppurative mastitis when they showed interstitial and/or alveolar mononuclear inflammatory cells (lymphocytes, plasmacells, macrophages) and mild to severe fibrosis; affected by chronic mixed mastitis when they showed interstitial and/or alveolar mononuclear inflammatory cells and a variable number of neutrophils; affected by acute suppurative mastitis when they showed interstitial and/or alveolar neutrophils (Fig. 1).

### Statistical analysis

Multinomial logistic regression performed using generalized linear mixed model (GLMM) was applied to evaluate the association among the histological findings, infection and animal attributes. The GLMM included as covariates the positivity for CNS, EO, PATO and SRLV to investigate relationship between infection status and histological lesions. The animal attributes included in GLMM were age and species which could be linked with histological lesions. Each analyzed udder was considered positive or negative for each investigated agent if one of its isolate showed positivity during laboratory tests.

A logistic regression, adjusted for animal age and presence of each bacteria class, was performed to investigate relationship between presence of follicles at histological examination and infections of SRLV. This model was adjusted for infection of bacterial groups (EO, CNS), age and species. The possible effect of age, species and SRLV positivity on colonization of udder by CNS, EO was tested with a series of logistic regressions performed using GLMM. Given the low number of udders showing positivity to PATO, it was not possible to perform such investigation for this class of bacteria. All regression models contained farm as random effect to adjust for sampling more individuals from same farm.

## Results

Among the 89 macroscopically healthy udders, 25/89 (28.09%; sheep = 6; goats = 19) were microbiologically negative, while at least one bacterial species was isolated in the remaining 64/89 (71.91%; sheep = 35; goats = 29). In the positive udders, 138 different bacterial species were isolated. The bacterial species isolated from udders of sheep and goats are reported in Table 1.

Coagulase-negative staphylococci (CNS) were the most prevalent bacteria isolated from both goats and sheep. Among the 138 isolated bacteria, 64 (46.42%) belonged to this group. The detail of the species isolated are summarized in Table 1.

Environmental opportunists were the second most frequently identified bacterial group (48/138; 34.76%). *Aerococcus viridans* was the predominant strain in both species being isolated in 29 samples and thus representing 21.01% of the total isolates. A total of 14/138 microorganisms (10.14%) were classified as “other microorganisms”. The most frequent microorganisms were *Acinetobacter lwoffii* and *Kocuria rosea*, with three cases each (2.17%). Pathogen bacteria were isolated in a lower percentage (12/138 samples; 8.68%). *Staphylococcus aureus* was found in 5 cases (3.62%), while *Streptococcus uberis* was identified in 4 (2.9%). *Streptococcus dysgalactiae*, *Pseudomonas aeruginosa* and *Escherichia coli* represented the 0.72% of isolated strains.

**Table 1:** Number and frequency of bacterial species isolated.

Microorganisms	Species	Isolates n°	%	Sheep	Goats
Pathogens					
	*Staphylococcus aureus*	5	3.62%	3	2
	*Streptococcus uberis*	4	2.90%	2	2
	*Streptococcus dysgalactiae*	1	0.72%	1	0
	*Pseudomonas aeruginosa*	1	0.72%	0	1
	*Escherichia coli*	1	0.72%	0	1
	Total of group	12	8.68%	6	6
Coagulase-negative staphylococci					
	*Staphylococcus arlettae*	2	1.46%	2	0
	*Staphylococcus auricularis*	5	3.62%	2	3
	*Staphylococcus capitis*	1	0.72%	0	1
	*Staphylococcus caprae*	7	5.07%	0	7
	*Staphylococcus chromogenes*	2	1.46%	2	0
	*Staphylococcus cohnii*	2	1.46%	1	1
	*Staphylococcus equorum*	7	5.07%	4	3
	*Staphylococcus haemolyticus*	2	1.46%	1	1
	*Staphylococcus kloosii*	1	0.72%	1	0
	*Staphylococcus lentus*	1	0.72%	1	0
	*Staphylococcus muscae*	2	1.46%	1	1
	*Staphylococcus sciuri*	2	1.46%	1	1
	*Staphylococcus simulans*	2	1.46%	1	1
	*Staphylococcus vitulinus*	4	2.90%	3	1
	*Staphylococcus warneri*	5	3.62%	3	2
	*Staphylococcus xylosus*	18	13.04%	9	9
	*Staphylococcus* spp.	1	0.72%	0	1
	Total of group	64	46.42%	32	32
Environmental opportunists					
	*Aerococcus viridans*	29	21.01%	19	10
	*Aeromonas hydrophila*	3	2.17%	3	0
	*Aeromonas sobria*	1	0.72%	1	0
	*Bacillus pumilus*	6	4.34%	1	5
	*Escherichia coli*	2	1.46%	1	1
	*Enterococcus cecorum*	1	0.72%	1	0
	*Enterococcus durans*	1	0.72%	0	1
	*Enterococcus faecalis*	1	0.72%	0	1
	*Klebsiella oxytoca*	2	1.46%	2	0
	*Pseudomonas fluorescens*	1	0.72%	0	1
	*Streptococcus constellatus*	1	0.72%	1	0
	Total of group	48	34.76%	29	19
Other microorganisms					
	*Acinetobacter lwoffii*	3	2.17%	1	2
	*Acinetobacter genomospecies*	1	0.72%	0	1
	*Acinetobacter* spp.	1	0.72%	0	1
	*Alcaligenes faecalis*	2	1.46%	2	0
	*Bordetella trematum*	1	0.72%	1	0
	*Kocuria rosea*	3	2.17%	3	0
	*Paenibacillus* spp.	1	0.72%	0	1
	*Vagococcus fluvialis*	2	1.46%	1	1
	Total of group	14	10.14%	8	6
Total		138	100%	75	63

Co-infections has been detected in 48/64 (75%) of the mammary glands; specifically, co-infections caused by two (42.2%) and three (26.5%) different bacterial species were demonstrated in 27/48 and 17/48 samples respectively. Four bacterial species (4.7%) were identified in 3/48 samples and five (1.6%) were found in 1/48 udder only. Only the 25% (16/64) of the positive udders were characterized by a single infection.

In four udders (4.5%) inhibiting substances were identified. Only one *Aspergillus* spp. (1.1%) grew on Sabouraud agar plate.

Small Ruminant Lentivirus infection was detected in 35/89 samples (39.3%). Most of them were isolated along with bacteria (21/35; 60%). Goats were more infected than sheep (29/35; 82.9% versus 6/35; 17.1%).

Histologically, 45/89 udders showed chronic non-suppurative mastitis (50.56%; sheep *n* = 17 and goat *n* = 28), 12 chronic mixed mastitis (13.48%; sheep *n* = 2 and goat *n* = 10), four acute suppurative mastitis (4.5%; sheep *n* = 1 and goat *n* = 3) and 28 did not show any lesions (31.46%; sheep *n* = 21 and goat *n* = 7).

In chronic mastitis the inflammatory cells were mainly located in the interstitium; alveolar neutrophils were detected in mixed (66%) or suppurative (100%) mastitis only. However necrosis and loss of the alveolar epithelium were never observed.

Microbiological and histological findings observed in the present study are reported in Table 2. In chronic non-suppurative mastitis, mixed mastitis and normal mammary glands, coagulase-negative staphylococci and environmental opportunists were the most frequently detected bacteria. On the contrary, acute suppurative mastitis showed a greater number of pathogenic microorganisms. Microbial agents were absent in 19/28 normal mammary glands only.

Within SRLV positive udders, 25/35 (71.5%) showed a chronic non-suppurative mastitis (Figs. 1A, 1B); 8/35 (22.9%) had a chronic mixed mastitis (Fig. 1C); 1/35 (2.8%) showed an acute suppurative mastitis (Fig. 1D) and only 1/35 (2.8%) did not show any lesions (Fig. 1E). Lentiviruses positive udders (68.6%; 24/35) revealed scattered hyperplastic lymphoid follicles throughout the parenchymal tissue and particularly around the lactiferous ducts (Fig. 1F).

Histological lesions were significantly associated to species and SRLV and CNS infections. Small Ruminant Lentivirus positive udders had significantly high risk to show both chronic mixed mastitis and chronic non-suppurative mastitis at histological examination (Table 3). Goats had significant higher risk to show chronic mixed mastitis compared to sheep (Table 3). CNS infection significantly increased the risk of chronic non-suppurative mastitis. Presence of PATO did not increase the risk of presenting one particular histological lesion (Table 3). Lentiviruses positive udders also showed a 12.3 (95% CI [3.8–45.2]; *p* < 0.01) times higher risk to have lymphoid follicles at histological examination. Goats had significant lower risk to be infected by EO compared to sheep (Table 4), but higher risk of being positive for SRLV (Table 5). Age and co-infections did not show any effects on bacterial or viral infections (Table 4).

**Table 2:** Summary of bacterial species identified within each histological type of mastitis.

Histological type of mastitis	Bacteriological data					Virological data
	PATO	CNS	EO	OM	Negative	SRLV
**Chronic non-suppurative mastitis**	6	34	22	8	13	25
Sheep	3	14	11	2	2	5
Goat	3	20	11	6	11	20
**Chronic mixed mastitis**	2	10	8	0	3	8
Sheep	1	2	3	0	0	0
Goat	1	8	5	0	3	8
**Acute suppurative mastitis**	3	1	2	0	1	1
Sheep	1	0	1	0	0	0
Goat	2	1	1	0	1	1
**Negative**	1	19	16	6	8	1
Sheep	1	16	14	6	4	1
Goat	0	3	2	0	4	0

**Notes.**

PATO Pathogens CNS Coagulase-negative staphylococci EO Environmental opportunists OM Other microorganisms SRLV Small Ruminant Lentiviruses

**Table 3:** Results from multinomial logistic regression applied to evaluate association among histological lesions in mammary gland of goat/sheep and bacterial infection, SRLV infection, age and species. Udders without sign of histological lesions were set as reference class for the multinomial regression.

Covariate	Chronic mixed mastitis [OR (95% CI)]	Chronic non-suppurative mastitis [OR (95% CI)]
Caprine (Ref: Ovine)	13.7 (1.75, 34.1)^*^	5.21 (0.83, 17.1)
Age (Ref: 1–3 years of age):		
4–8 years of age	3.14 (0.46, 22.65)	2.21 (0.74, 12.19)
>8 years of age	1.32 (0.14, 10.46)	0.82 (0.15, 4.42)
EO	2.61 (0.46, 17.16)	2.18 (0.41, 4.79)
CNS	7.25 (0.59, 22.10)	6.72 (1.83, 28.51)^*^
PATO	9.21 (0.68, 25.67)	7.8 (0.56, 16.71)
SRLV	47.5 (3.15, 80.16)^**^	43.52 (4.30, 104.52)^**^

**Notes.**

* *p* < 0.05.

** *p* < 0.01.

EO Environmental opportunists CNS Coagulase-negative staphylococci PATO Pathogens SRLV Small Ruminant Lentivirus

**Table 4:** Results from logistic regressions applied to evaluate factors in mammary gland of sheep/goat which can favour infection of CNS and EO.

Covariate	CNS [OR (95% CI)]	EO [OR (95% CI)]
Caprine (Ref: Ovine)	0.12 (0.32, 4.2)	0.26 (0.06, 0.71)^*^
Age (Ref: 1–3 years of age):		
4–8 years of age	1.25 (0.61, 3.32)	1.27 (0.27, 3.805)
>8 years of age	1.34 (0.48, 5.76)	2.09 (0.38, 7.45)
EO	2.16 (0.79, 4.98)	–
CNS	–	3.51 (0.59, 5.31)
PATO	1.23 (0.21, 4.67)	2.31 (0.42, 6.34)
SRLV	1.52 (0.42, 3.78)	2.97 (0.60, 4.24)

**Notes.**

* *p* < 0.05.

** *p* < 0.01.

EO Environmental opportunists CNS Coagulase-negative staphylococci PATO Pathogens SRLV Small Ruminant Lentivirus

**Table 5:** Results from logistic regressions applied in mammary gland of goat/sheep to evaluate factors that can favour SRLV infection.

Covariate	CNS [OR (95% CI)]
Caprine (Ref: Ovine)	10.87 (3.69, 37.77)^**^
Age (Ref; 1–3 years of age):	
4–8 years of age	0.66 (0.18, 2.33)
>8 years of age	0.46 (0.11, 1.82)
EO	1.67 (0.41, 3.29)
CNS	1.15 (0.58, 5.18)
PATO	0.67 (0.34, 4.89)

**Notes.**

* *p* < 0.05.

** *p* < 0.01.

EO Environmental opportunists CNS Coagulase-negative staphylococci PATO Pathogens

## Discussion

This is the first study investigating the presence of microbial agents and histological features in macroscopically healthy mammary glands of small ruminants.

Coagulase-negative staphylococci prevail, (64/138; 46.42%), in agreement with the literature. Already Bergonier et al. (2003b) reported a CNS prevalence in subclinical mastitis ranging from 25 to 93%. Similar results (42.9%) were recently observed also in Portugal in milk samples from clinically affected sheep udders (Queiroga, 2017), in Iran in subclinical ovine mastitis (Narenji Sani, Mahdavi & Moezifar, 2015), in Greece in dairy goats (Gelasakis et al., 2016) and in Italy in both species (Dore et al., 2016). The identification of *Staphylococcus xylosus* in sheep and *Staphylococcus caprae* in goats is also in agreement with the literature (Bergonier et al., 2003b; Contreras et al., 2007; Ergün et al., 2009), while *Staphylococcus equorum* was isolated only by Hariharan et al. (2004) in sheep and by Contreras et al. (2003) in goats. Differently from previous studies reporting a common isolation (Bergonier et al., 2003b; Contreras et al., 2007), *Staphylococcus chromogenes* was observed in two samples only. According to the literature (Contreras et al., 2007; Gelasakis et al., 2015), the other species of CNS identified in the present research were observed with a lower frequency (Table 1).

The role of OM in the epidemiology of small ruminants mastitis is still unclear. Only *Aerococcus viridans* and *Enterococcus* spp. have previously been described as a sporadic cause of mammary infection in sheep and goats (Clements, Taylor & Fitzpatrick, 2003; Marogna et al., 2012; Queiroga, 2017).

Goats had a significant lower risk to be infected by EO compared to sheep. Since EO prevalence increases when deficiencies in mechanical milking system or milking hygiene in small ruminants flocks (particularly in sheep flocks) occur, milking routines as well as milking equipment must be improved and periodically checked.

The low rate of fungi isolation *Aspergillus* spp. (1.1%) can be related to their common detection in clinical mastitis (Bergonier et al., 2003b), while the present study reports the isolation in macroscopically unaffected tissues.

The SRLV identification (35/89; 39.3%) agrees with the literature (Fras et al., 2013). Our work also showed that SRLV were more likely detected in goat udders than sheep (29/35; 82.9% versus 6/35; 17.1%). The high prevalence of SRLV infection in goats could be caused by the horizontal transmission with colostrum and milk. Indeed, in sheep industry, lambs usually suckle milk from their dam. On the contrary, kids are separated from mothers at birth, housed together, and hand-fed colostrum and milk, often from a common pool. So, a SRLV presence in the mammary tissue of a single animal may infect all the young animals. This occurrence has been reported to be the most reliable reason for the high prevalence of CAEV infection in goats in Western Europe, Australia, and North America (Narayan & Cork, 1985; Fras et al., 2013).

Small Ruminant Lentivirus infections have been associated with a spectrum of immune-mediated diseases and opportunistic infections (Brodie et al., 1995). In goats, several studies observed that SRLV infected animals appear to be more predisposed to subclinical bacterial infections by non-haemolytic staphylococci (Smith & Cutlip, 1988; Ryan, Greenwood & Nicholls, 1993). Also in the present study, goats showed a significant higher risk to show chronic mixed mastitis compared to sheep.

In sheep, *in vivo* relationship between SRLV and bacteria is not known, but previous *in vitro* studies provided evidence for increased bacterial adherence and decreased phagocytosis in macrophages infected by SRLV (Monleón et al., 1997). In the present study, chronic mastitis observed in SRLV positive udders is frequently associated with CNS and EO infections. Although the SRLV positive samples showed a significantly higher risk to be affected by both mixed and non-suppurative mastitis, co-infections did not show any effects on the virus detection.

Despite the udders considered in the present study being macroscopically healthy, the absence of lesions at the histological screening was observed in 28 samples only. The remaining 61 udders showed different types of mastitis; most of them were chronic (64%) and mild in severity. Mild to severe fibrosis was observed in all the samples (28%: slight; 25.8%: moderate; 46%: severe). However, this finding cannot be related to the chronicity of the disease only, because the ratio between parenchyma and stroma shows individual proportions and changes during the different phases of the reproductive cycle of the animals and of the lactation cycle (Lérias et al., 2014). The mastitis chronicity was mainly evaluated on the basis of the inflammatory cells type, diagnosing a chronic mastitis when the exudate was composed by macrophages, histiocytes and plasmacells. An interesting finding is represented by the identification of these non-suppurative cells mainly in the interstitium; alveolar and ductal cells (neutrophils) were detected in mixed or suppurative mastitis only. In these udders, an increased number of desquamated cells was also observed in the alveoli.

The correlation between microbiological and histological findings suggests that the animals in which CNS were isolated had a significantly increased risk to develop a chronic non-suppurative mastitis. There were no other significant correlations. However, in acute suppurative mastitis pathogen bacteria were always isolated, except in one case in which the identification of inhibiting substances justifies a negative microbiological examination (Zavanella, 2011).

At histological examination, SRLV positive udders revealed scattered hyperplastic lymphoid follicles. Indeed, SRLV generally causes induritive and non-suppurative mastitis characterized by periacinar interstitial diffuse mononuclear cell infiltration, interstitial intense fibrosis and hyperplastic lymphoid follicle formation scattered throughout the parenchymal tissue particularly around the lactiferous ducts (Van der Molen, Vecht & Houwers, 1985; Brodie et al., 1995; Monleón et al., 1997). Eleven animals affected by chronic non-suppurative mastitis and bacteriologically negative were positive for SRLV, thus confirming the role of SRLV in subclinical chronic mastitis (Plummer & Plummer, 2012). The statistical model results further support this result.

## Conclusions

The results of the present study demonstrate that macroscopically unaffected glands of small ruminants do not represent a sterile substrate, but may be a reservoir of microbial agents whose role remains to be better evaluated. The detection of a large number of bacterial species improves the current knowledge of the species capable of colonizing and potentially infecting mammary glands of small ruminant. Many bacteria are able, at least temporarily, to be sequestered inside the mammary tissue. This ability might be one of the mechanisms that these bacterial species adopt to by-pass host defenses and treatments, allowing them to persist within individuals and the flock.

Therefore, from a flock management perspective, small ruminants with subclinical chronic mastitis have a potential risk to develop acute or chronic clinical disease. Given the paucity of the sample size of our data, the magnitude of risk cannot be determined from the current study. The high number of EO and OM suggests the need to develop and apply procedures for controlling microbial and environmental contamination and consequently to improve hygiene and quality of milk and dairy products and finally the consumers’ welfare.

Despite some histological lesions being associated with SRLV and CNS infections (hyperplastic lymphoid follicles and non-suppurative mastitis), it seems difficult to correlate each histological pattern with specific microbial isolation.

Possible future investigations could aim to screen macroscopically affected mammary glands in order to evaluate the relationships between the isolated biological agents in clinical acute and chronic mastitis with histological patterns.

##  Supplemental Information

10.7717/peerj.3994/supp-1 Data S1 Raw data

## Additional Information and Declarations

### Data Availability

The following information was supplied regarding data availability: The raw data has been provided as Data S1.
